# Loss of Sonic Hedgehog Leads to Alterations in Intestinal Secretory Cell Maturation and Autophagy

**DOI:** 10.1371/journal.pone.0098751

**Published:** 2014-06-02

**Authors:** Jessica Gagné-Sansfaçon, Joannie M. Allaire, Christine Jones, François Boudreau, Nathalie Perreault

**Affiliations:** Département d’Anatomie et Biologie Cellulaire, Faculté de Médecine et des Sciences de la Santé, Université de Sherbrooke, Sherbrooke, QC, Canada; University of Florida, United States of America

## Abstract

**Background:**

Intestinal epithelial cells express the Sonic and Indian hedgehog ligands. Despite the strong interest in gut hedgehog signaling in GI diseases, no studies have specifically addressed the singular role of intestinal epithelial cell Sonic hedgehog signaling. The aim of this study was to investigate the specific role of Sonic hedgehog in adult ileal epithelial homeostasis.

**Methodology/Principal Findings:**

A Sonic hedgehog intestinal epithelial conditional knockout mouse model was generated. Assessment of ileal histological abnormalities, crypt epithelial cell proliferation, epithelial cell fate, junctional proteins, signaling pathways, as well as ultrastructural analysis of intracellular organelles were performed in control and mutant mice. Mice lacking intestinal epithelial Sonic Hedgehog displayed decreased ileal crypt/villus length, decreased crypt proliferation as well as a decrease in the number of ileal mucin-secreting goblet cells and antimicrobial peptide-secreting Paneth cells during adult life. These secretory cells also exhibited disruption of their secretory products in mutant mice. Ultrastructural microscopy analysis revealed a dilated ER lumen in secretory cells. This phenotype was also associated with a decrease in autophagy.

**Conclusions/Significance:**

Altogether, these findings indicate that the loss of Sonic hedgehog can lead to ileal secretory cell modifications indicative of endoplasmic reticulum stress, accompanied by a significant reduction in autophagy.

## Introduction

Morphogens are soluble molecules which form patterning gradients in tissues [1] and play key roles in adult tissue and cell homeostasis. Hedgehog ligands (Hh) are secreted multifunctional morphogens regulating developmental and cellular processes including tissue homeostasis and repair, cell survival and proliferation in the gastrointestinal (GI) tract [1], [2]. Intestinal epithelial cells express Hh ligands, such as Sonic hedgehog (Shh) in crypt cells and Indian hedgehog (Ihh) in villous cells [1]. Secreted Hh ligand stimulation of cells expressing the Patched receptor (Ptc1) leads to the downstream activation of the Smoothened co-receptor and of Gli transcription factors [1]. Although closely related, both hedgehog ligands display phenotypic differences when genetically abrogated in mice. The ubiquitous inactivation of Hh ligands results in specific gut phenotypes in embryonic and neonatal mice. *Shh* mutants exhibit anterior expansion of the glandular stomach, increased gland fission, duodenal obstruction and abnormal innervation of the gut in addition to expressing certain markers reminiscent of early intestinal transformation of the stomach [1], [3] whereas *Ihh* mutants exhibit reduced epithelial stem cell proliferation and differentiation [4]. Based on these data, it was assumed that Hh ligands produced by intestinal epithelial cells could act on the mesenchyme through paracrine signaling, thereby inducing mesenchymal signals including Secreted-frizzled-related proteins (SFRP1 and 2) and Bone morphogenetic proteins (Bmps) affecting intestinal epithelial cell proliferation as well as differentiation by antagonizing Wnt signaling [2], [5]–[9]. However, additional evidences have suggested that an autocrine canonical and non-canonical Hh signaling pathway, occurring in the crypt intestinal stem cell region, is also important for gut homeostasis [10]–[12]. Indeed, both intestinal stem cells and mature mesenchyme surrounding intestinal crypts have been shown to respond to Hh-Gli signaling.

The intestinal epithelium represents a dynamic system in perpetual renewal [13], [14]. The adult intestinal mucosa is comprised of both undifferentiated and pluripotent stem cells, located in the lower portion of the intestinal crypt, as well as differentiated and functional epithelial cells found along the villus axis. Terminally differentiated intestinal epithelial cells (IECs) derived from stem cells are divided into absorptive cells, which play a role in the absorption of nutrients, and into cells of the secretory lineage which include mucin-secreting goblet cells, hormone secreting-enteroendocrine cells, and antimicrobial peptide-secreting Paneth cells [15]. Small intestinal epithelial homeostasis including crypt/villus architecture, cell proliferation, differentiation and apoptosis are spatially and temporally regulated by a number of signaling pathways [15].

Despite the strong interest in gut Hh signaling in GI diseases [2], [7], [16]–[20], no studies have specifically addressed the singular role of IEC Shh signaling. By using specific IEC conditional knockout mice, we have uncovered an important role for Shh in ileal goblet and Paneth cell function. Results demonstrate that deficiency in Shh can lead to Paneth secretory cell modifications indicative of endoplasmic reticulum (ER) stress, accompanied by a significant reduction of the autophagic process. These observations identify Shh signaling as a potential environmental modulator of IEC autophagy as well as an important biological process for IEC secretory cell function [21] and ileal tissue homeostasis [21]–[23].

## Materials and Methods

### Animals

129SvEv-*Shh* ^fx/fx^ mice were purchased from Jackson Laboratory (stock #004293) while the mouse C57BL/6 12.4 KbVilCre transgenic line was kindly provided by Dr. Gumucio [24]. Genomic DNA was isolated using the Spin Doctor genomic DNA kit from Gerard Biotech following the manufacturer’s protocol. All mutations were genotyped according to already published protocols [24]. All experiments were approved by the Animal Research Ethics Committee of the Faculty of Medicine and Health Sciences of the Université de Sherbrooke (approval ID number 114-11B).

### Tissue Preparation and Histological Staining

Digestive tracts from 180 to 210 day-old *Shh* ^ΔIEC^ mice and control littermates were fixed, sectioned and stained (H&E and Alcian blue) as previously described [25]–[28].

### Tissue Collection, RNA Extraction and Gene Expression Analysis

RNA from scraped intestinal mucosal was isolated and processed using the Totally RNA extraction kit (Ambion). Reverse-transcription PCR (RT-PCR) and quantitative real-time PCR were performed as described previously [25]–[28]. PCR conditions and primers sequences are available upon request.

### Cell Fractionation along the Crypt-villus Axis

C57BL/6 wild-type mice obtained from Charles River Laboratories (Wilmington, MA) were sacrificed and the small intestine harvested, inverted onto polyethylene tubing, ligatured at both extremities, and washed with KRB buffer, pH 7.5, as described previously [29]. Segments were then incubated under agitation in ice-cold isolation buffer (2.5 mM EDTA, 0.25 mM NaCl) for 2-min intervals. After each interval, cell suspensions were centrifuged at 400 × g for 5 min. Pellets were then washed with ice-cold KRB buffer and lysed for total RNA isolation [29].

### Immunostaining and TUNEL Assay

Immunofluorescence and immunohistochemistry staining were performed as previously described [25]–[28]. Non-specific binding was blocked and antibodies were diluted in PBS/Triton 0.1% solution containing 2% BSA (Sigma-Aldrich). The following antibodies were used at the indicated dilutions: anti-PCNA (1∶1000, Abcam), anti-lysozyme (1∶800, DakoCytomation), anti-UEA-1 (1∶1000, Sigma), anti-chromogranin A (1∶1000, Immunostar), anti-E-cadherin (1∶1000, BD), anti- β-catenin (1∶500, Cell Signaling), anti-IFABP (1∶500, kindly provided by Dr. Gordon [30]) and FITC-conjugated anti-rabbit IgG (1∶200, Santa Cruz). TUNEL assay was performed according to the manufacturer’s protocol (Roche Diagnostic).

### Protein Extraction and Western Blot Analysis

Total proteins were isolated from scraped intestinal mucosa of 180 to 210 day-old *Shh* ^ΔIEC^ mice and control littermates with RIPA buffer (50 mM Tris pH 7.4, 150 mM NaCl, 1% NP40, 0.5% Triton X-100, 1 mM EDTA, 0.2% SDS, 0.5% Na-deoxycholate) containing protease and phosphatase inhibitors [31]. Thirty µg of protein extract were analyzed by 10% BisTris NuPAGE (Invitrogen) and transferred onto a PVDF blotting membrane (Roche Diagnostics, QC), after which Western blotting was performed as described [25], [32]. The following affinity-purified antibodies were used: claudin-1 rabbit polyclonal antibodies (1∶500), claudin-2 rabbit polyclonal antibodies (1∶1000), occludin rabbit polyclonal antibodies (1∶500), JAM-A rabbit polyclonal antibodies (1∶500) from Zymed Laboratories (Invitrogen); LC3b-I and LC3b-II rabbit polyclonal antibodies (1∶1500), E-cadherin rabbit polyclonal antibodies (1∶1000), IRE1α rabbit polyclonal antibodies (1∶500) from Cell Signaling; p62 mouse monoclonal antibodies (1∶1000) from Abcam, and actin (1∶10 000), defensin 4 goat polyclonal antibodies (1∶250), c-Myc rabbit polyclonal antibodies (1∶200) and Cyclin D1/D2 rabbit polyclonal antibodies (1∶500) from Santa Cruz. For densitometry analyses, exposed films of Western blots were scanned and images were analyzed using ImageJ (Rasband, WS, ImageJ, US National Institutes of Health, Bethesda, Maryland, USA).

### Electron Microscopy

Portions of mouse ileal segments were rinsed with PBS, prefixed for 15 min with a 1∶1 mixture of culture medium (Dulbecco’s modified Eagle’s medium) and freshly prepared 2.8% glutaraldehyde in cacodylate buffer (0.1 M cacodylate and 7.5% sucrose), then fixed for 30 min with 2.8% glutaraldehyde at room temperature. After two rinses, specimens were post-fixed for 1 h with 2% osmium tetroxide in cacodylate buffer. The tissues were then dehydrated using graded ethanol concentrations (40, 70, 90, 95 and 100%, three times each) and coated twice for 3 h with a thin layer of Araldite 502 resin (for ethanol substitution). Finally, the resin was allowed to polymerize at 60°C for 48 h. The specimens were detached from the plastic vessels, inverted in embedding molds, immersed in Araldite 502, and polymerized at 60°C for 48 h. Ultramicrotome-prepared thin sections were contrasted with lead citrate and uranyl acetate, and subsequently observed on a Jeol 100 CX transmission electron microscope. All reagents were purchased from Electron Microscopy Sciences (Cedarlane, Hornby, ON, Canada).

### Quantification and Statistical Analyses

All histological and cell count analyses or scores were performed in a blinded manner using continuous sections from low-powered fields of well-oriented intestinal cross-sections on an average of 10 independent fields per animal. Variations in normalized-numbers of goblet and enteroendocrine cells and in mean numbers of Paneth cells per villus were calculated as previously described [25]–[27], [33]. Briefly, the total number of goblet and enteroendocrine cells were counted per crypt-villus axis and normalized by the total number of nuclei. Nuclei were counted following 4′,6-diamidino-2-phenylindole (DAPI) staining. Paneth cells were quantified with lysozyme immunofluorescence. Images of lysozyme-labeled Paneth cells were acquired and positive cells were counted per crypt/villus axis. Statistical analysis was performed using two-way ANOVA. Differences were considered significant with a *p* value of <0.05. All statistical analyses were carried out using Graph Pad Prism 5 (Graph Pad Inc, San Diego, CA).

## Results

### Loss of Intestinal Epithelial Shh Decreases Small Intestinal Length and Epithelial Cell Proliferation

**Figure 1:**
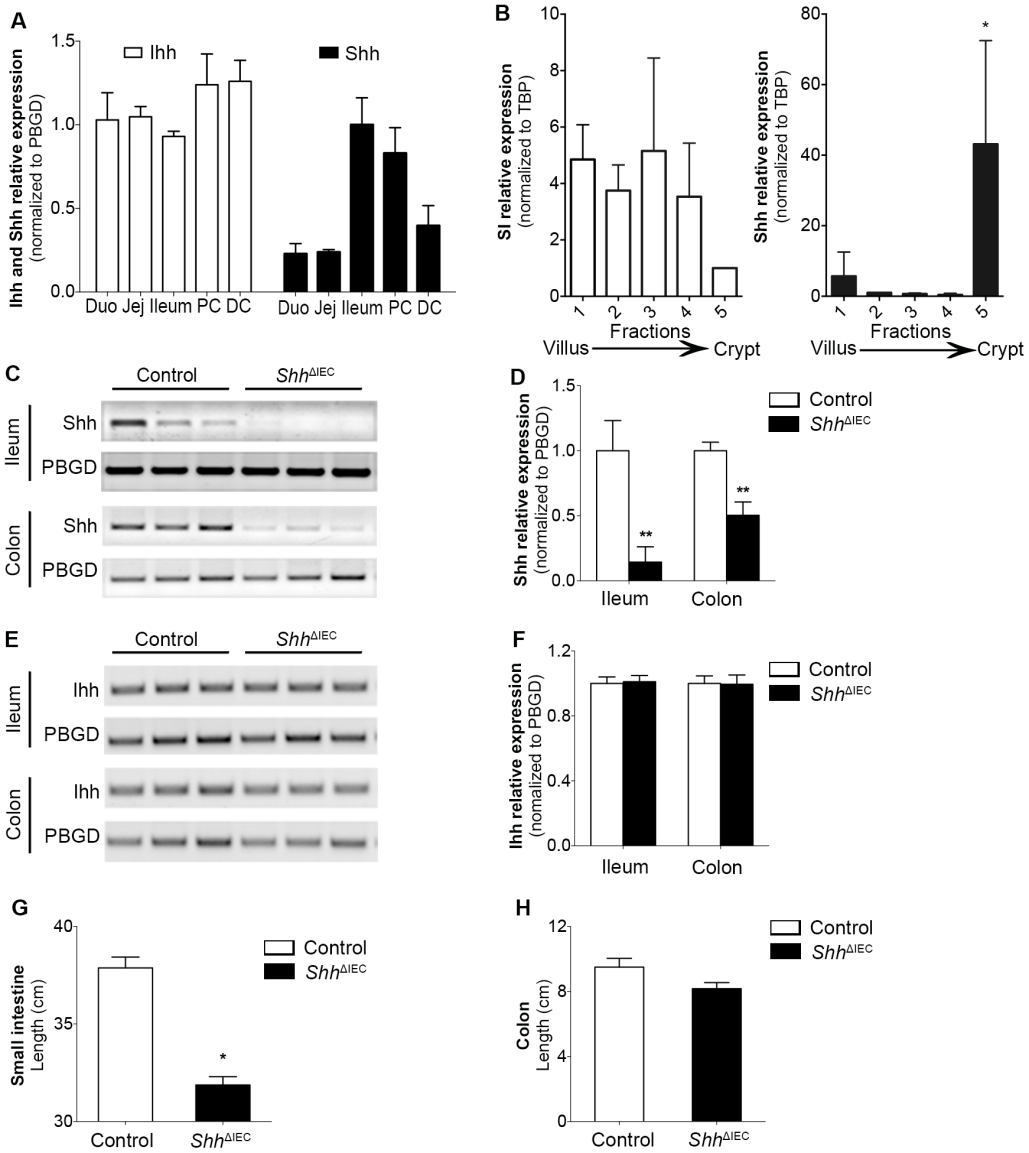
Shh and Ihh expression levels along the intestinal rostro-caudal axis and loss of Shh expression in the ileal and colonic epithelium of *Shh* ^ΔIEC^ mice. Quantification of Ihh and Shh mRNA expression levels along the intestinal rostro-caudal axis was performed by qPCR (A) (n = 7). Total mRNA from intestinal epithelial fractions isolated along the villus-to-crypt axis by the Weiser method (n = 3) were used to monitor mRNA levels of sucrase-isomaltase and Shh along the crypt-to-villus axis (B). Semi-quantitative PCR analysis was performed to detect Shh mRNA expression levels in the ileum and colon of *Shh* ^ΔIEC^ and control mice (C and D). Semi-quantitative PCR analysis showed no modulation in Ihh mRNA expression levels in *Shh* ^ΔIEC^ mice when compared with control animals (E and F). Statistical analysis indicated a significant 1.2-fold decrease in small intestine length of 180-day-old *Shh* ^ΔIEC^ mice when compared to controls (G) whereas no modulation was observed in the colon (H) (n = 7). One-way ANOVA *p<0.05 (B), Student T-test *p<0.05, **p<0.01 (D and G). Error bar represent SEM. SI, sucrase-isomaltase; Duo, duodenum; Jej, jejunum, PC, proximal colon; DC, distal colon.

Ihh is the main Hedgehog expressed in the small and large intestine [1], [5], [8], [17], [18]. It has been suggested that low levels of Shh may potentially be expressed at the base of small intestinal and colonic crypts although such presence remains controversial [1], [8]. PCR analyses were therefore performed to confirm the distribution of Shh and Ihh expression along the rostro-caudal axis of the adult murine intestine. Shh was found to be expressed at its highest levels in the ileum and proximal colon whereas Ihh was abundantly and constantly detected along the entire intestinal tract (Figure 1A). To investigate the profile of Shh ligand expression in epithelial cells, epithelial populations from the mouse intestinal mucosa were progressively isolated along the villus-to-crypt axis using a modified Weiser procedure [29]. Total RNA was isolated from these epithelial cell populations, and Shh gene transcript levels were assessed by RT-qPCR analyses. Shh mRNA expression was significantly detected in the fraction corresponding to the lower part of the crypt (fraction #5) along the villus-to-crypt axis in contrast to the induction of the sucrase-isomaltase gene transcript in the villus epithelial cell fractions, a specific marker of intestinal epithelial cell differentiation (Figure 1B). Based on this expression profile, we then hypothesized that Shh could play a specific role in adult distal ileal homeostasis. Homozygous floxed *Shh* mice (*Shh* ^fx/fx^) were thus cross-bred with the 12.4 Kb*Vil*Cre transgenic line, which exclusively directs expression of the transgene in all intestinal epithelial cells, including stem cells [24]. Conditional knockout mice for *Shh* (*Shh* ^ΔIEC^) were born at the expected Mendelian ratios, and grew normally without obvious gross physical abnormalities. Comparative analysis of Shh and Ihh mRNA expression between control and *Shh* ^ΔIEC^ mice confirmed an 86% reduction in Shh expression in the ileum and a partial 50% reduction in the colon of mutant mice when compared to controls (Figures 1C and D). Importantly, no compensatory change in Ihh expression was observed in *Shh* ^ΔIEC^ mice (Figures 1E and F). Analysis showed that small intestine length in mutant animals was consistently shorter when compared to control littermates (Figure 1G) whereas colon length appeared not to be macroscopically affected by the partial loss of epithelial *Shh* (Figure 1H). Statistical analysis indicated a significant decrease of 1.2-fold in length of the small intestine in mutant mice when compared to control littermates.

**Figure 2:**
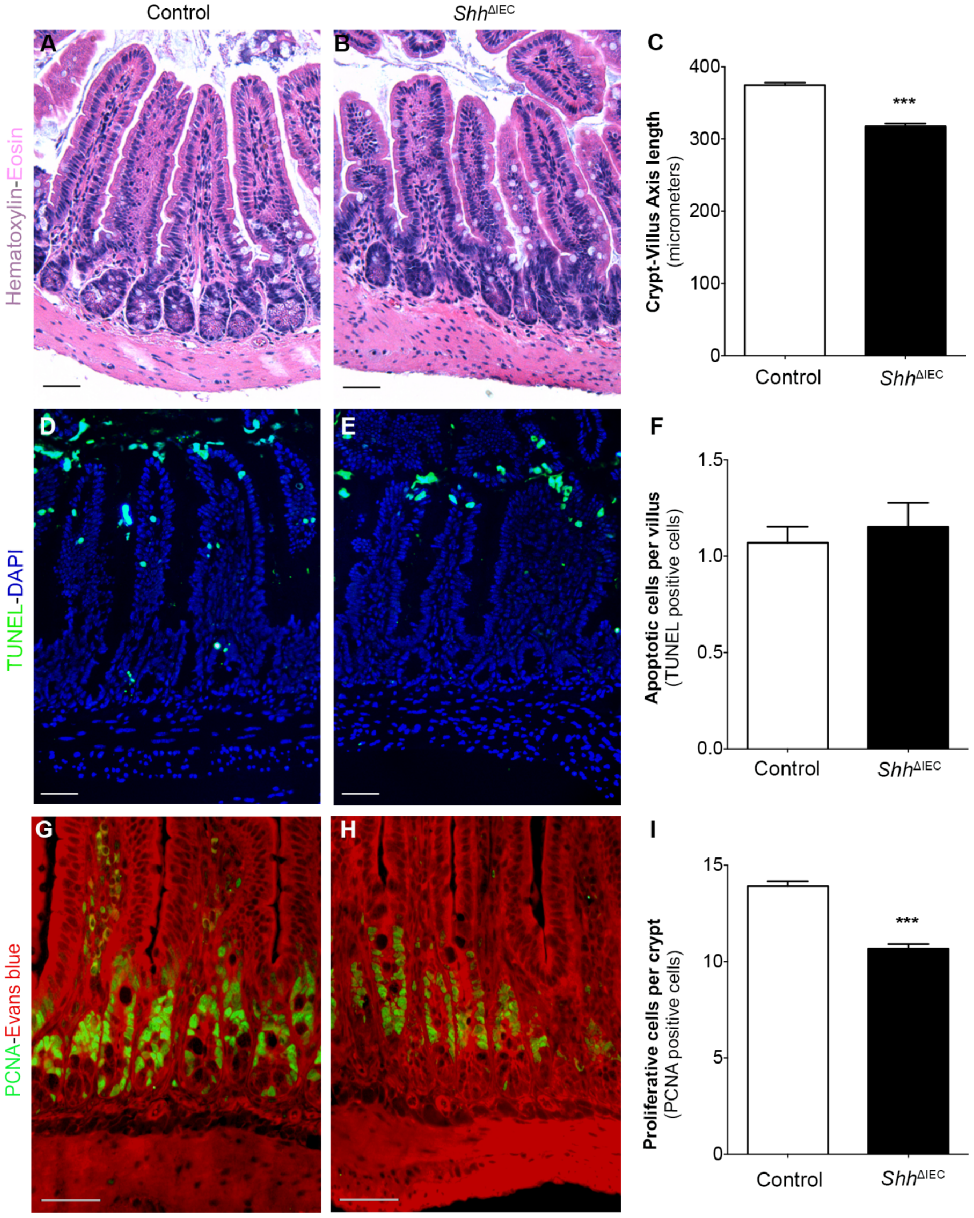
Loss of intestinal epithelial Shh signaling deregulates intestinal crypt epithelial proliferation. Hematoxylin and eosin staining was performed on ileal paraffin sections of 180-day-old control (A) or *Shh* ^ΔIEC^ (B) mice. The length of the crypt/villus axis was determined using MetaMorph v7.7 software and statistical analysis revealed a 1.2-fold reduction in crypt-villus axis length in *Shh* ^ΔIEC^ ileum when compared to controls (C) (n = 4). Apoptosis assays by TUNEL staining were performed on paraffin sections of 180-day-old control (D) or *Shh* ^ΔIEC^ (E) mice. DAPI (blue staining) served as a counterstain. No modulation in the number of TUNEL-positive cells was observed between mutant and controls (F). Proliferation assays were performed by PCNA immunostaining (G and H, green labeling), with Evans blue serving as counterstain (red staining). Mutants displayed a decrease in cell proliferation as shown by a decrease in the number of PCNA-labeled proliferating cells (H) when compared to controls (G). The number of PCNA-positive cells was quantified in the ileum of both controls and mutants (n = 4). Statistical analysis of the number of positive PCNA cells revealed a significant 1.3-fold decrease in proliferation in mutant animals (I). (n = 4) Two-way ANOVA ***p<0.001. Scale bar: 50 µm. Error bars represent SEM.

We next focused on histological analysis of *Shh* ^ΔIEC^ intestine. While most of the intestinal segments did not show significant histological changes in *Shh* ^ΔIEC^ mice (data not shown), the ileum of mutant mice exhibited a shorter crypt/villus axis (Figure 2B) when compared to control littermates (Figure 2A). Measurement of the ileal crypt/villus length in *Shh* ^ΔIEC^ and control littermates revealed a significant 1.2-fold decrease (Figure 2C). We next analyzed whether proliferation and/or programmed cell death were altered in these mice. TUNEL immunostaining revealed no significant difference in the number of apoptotic cells in the proximal to distal intestinal epithelium of *Shh* ^ΔIEC^ mice when compared to controls (Figures 2D–F). Although no significant change was observed in the jejunum of *Shh* ^ΔIEC^ mice (data not shown), ileal PCNA-labeling showed a significant 1.3-fold decrease in the number of proliferating cells in *Shh* ^ΔIEC^ mice when compared to controls (Figures 2G–I). These results indicate that the shortening of the ileal crypt/villus axis may be dependent on a deregulation in the epithelial cell proliferation program.

**Figure 3:**
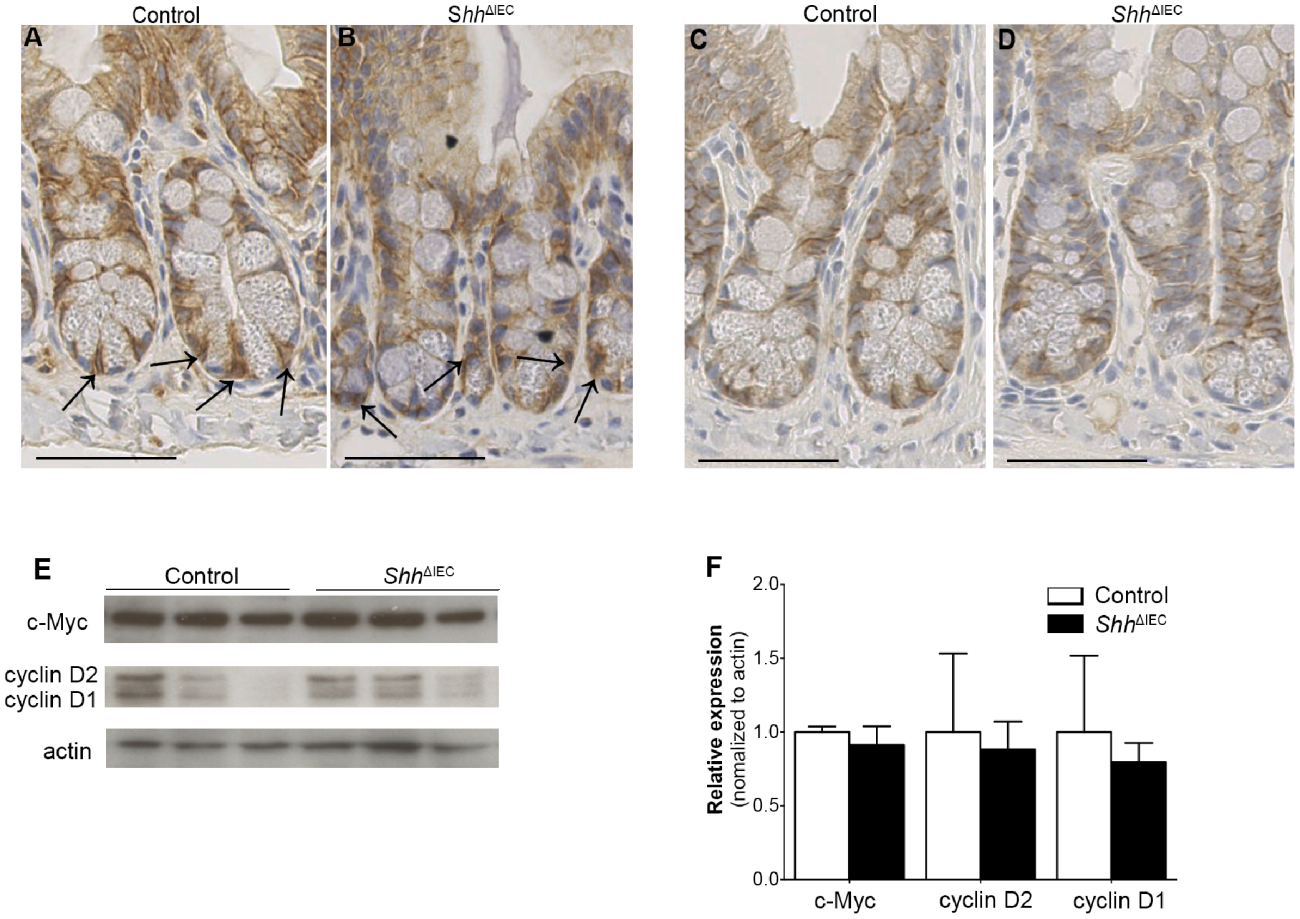
Loss of intestinal epithelial Shh signaling does not activate the Wnt/β-catenin pathway. Immunostaining with an anti-β-catenin antibody revealed no modulation of nuclear translocation of β-catenin (black arrows) between controls (A) and mutant mice (B). Immunostaining with an anti-E-cadherin antibody in both control (C) and mutant mice (D) enabled to determine the localization of the cell membrane relative to that of the nucleus. Western blot analysis for c-Myc and cyclin D1/D2 classical targets of epithelial cell proliferation was performed on ileal extracts from control and *Shh* ^ΔIEC^ mice (E). Densitometry analysis of exposed films using ImageJ revealed no significant modulation in c-Myc and cyclin D1/D2 expression levels in *Shh* ^ΔIEC^ mice compared to controls (F) (n = 3) Student t-test. Scale bar: 50 µm.

Given that, in previous studies, Ihh was shown to modulate intestinal epithelial proliferation via the Wnt/β-catenin pathway [5], [18], we thus investigated if deregulation of intestinal epithelial proliferation in *Shh* ^ΔIEC^ mice could similarly be dependent on an alteration of the Wnt/β-catenin pathway. No modulation in nuclear labeling of β-catenin was observed in *Shh* mutants (Figure 3B) compared to controls (Figure 3A). E-cadherin labeling in both control and mutant mice (Figures 3C and D, respectively) enabled to determine the localization of the cell membrane relative to that of the nucleus. Also analyzed were the expression levels of the proto-oncogene c-Myc, identified as a direct and required target gene of Wnt/β-catenin signaling [34], as well as cyclin D1/D2, another classical target of Wnt/β-catenin signaling. Western blot analysis on total ileal extracts revealed no significant modulation in c-Myc and cyclin D1/D2 expression levels between mutant and control mice (Figures 3E and F).

### Absorptive and Enteroendocrine Cell Differentiation are not Affected by the Loss of Intestinal Epithelial Shh Signaling

**Figure 4:**
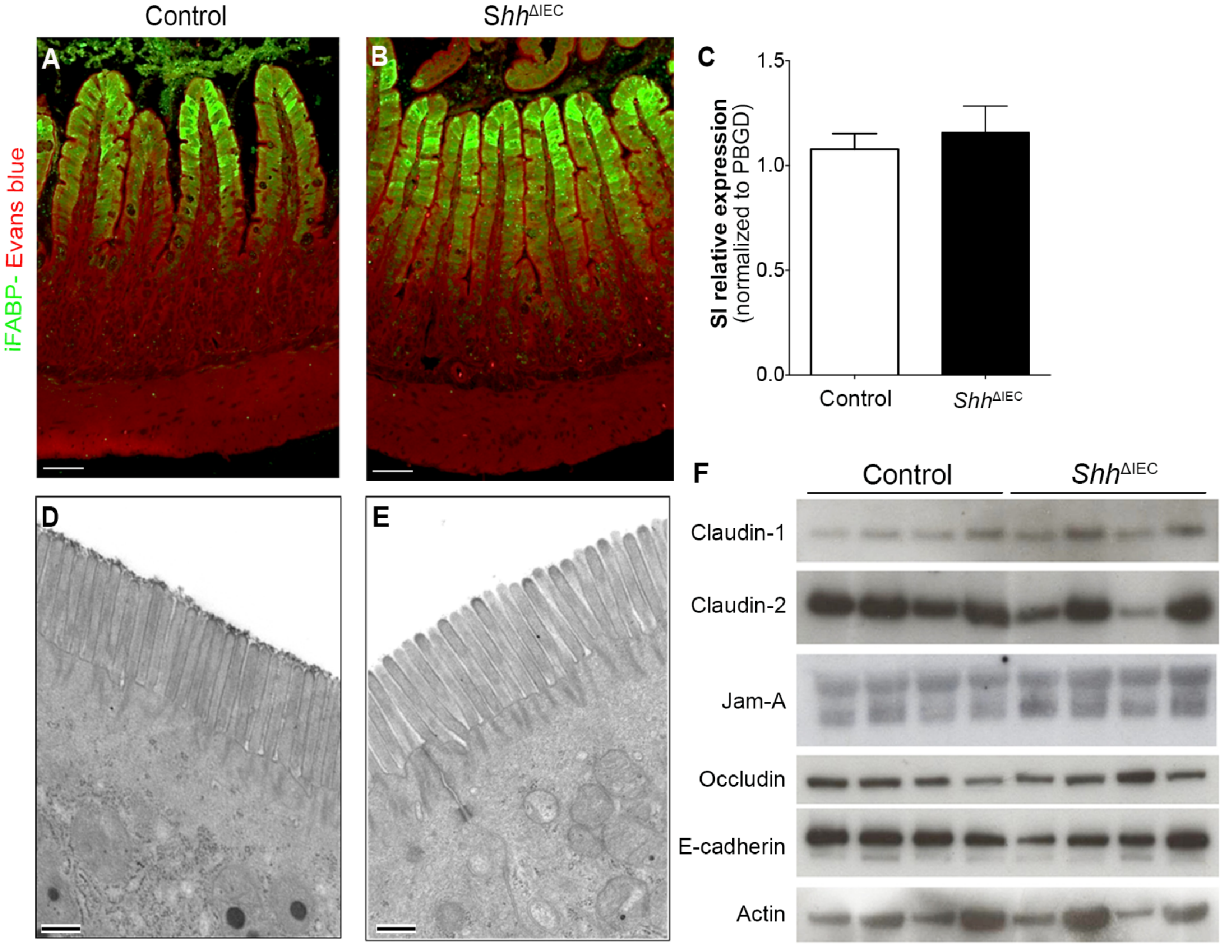
Absorptive cell differentiation is not affected by the loss of intestinal epithelial Shh signaling. Immunostaining for intestinal fatty acid binding protein (iFABP) (green labeling) was performed on 180-day-old *Shh* ^ΔIEC^ mice (B) and control littermates (A). Evans blue (red staining) served as a counterstain. Quantitative RT-PCR of sucrase-isomaltase revealed no modulation between mutant and control mice (C) (n = 7). Ultrastructural assessment of the apical membrane of ileal enterocytes from mutant mice (E) showed normal apical brush border and junctional complexes when compared to control littermates (D). Western blot analysis of claudin-1 and 2, Jam-A, occludin and E-cadherin was performed on total ileum lysates isolated from *Shh* ^ΔIEC^ and control mice (F) (n = 4). Scale bar: 50 µm (A and B). Scale bar: 0.5 µm (D and E) Error bars represent SEM. SI, sucrase-isomaltase.

We next investigated whether absorptive cell differentiation could be affected in the ileum of *Shh* mutant mice. Differentiated cells of the absorptive lineage express specific markers such as intestinal fatty acid binding protein (iFABP) and sucrase-isomaltase (SI) [35], [36]. Immunostaining for iFABP (Figures 4A and B) and quantitative RT-PCR for SI (Figure 4C) revealed no modulation in the expression of these markers. Furthermore, no ultrastructural modification of the apical brush border was noted between absorptive cells from control and mutant mice (Figures 4D and E). Maintenance of gut epithelial barrier integrity involves tight and adherent junctions (TJ and AJ, respectively), a characteristic feature of functional absorptive cells [37], [38]. Alterations in the expression of junctional proteins impair the functionality of these junctions [39], [40]. To analyze any modulation in the proteins constituting the TJ and AJ, Western blot analysis of E-cadherin, occludin, claudin-1, claudin-2 and JAM-1 were performed on total ileal mucosal extracts harvested from mutant and control mice (Figure 4F). No modulation was observed for any of the junctional proteins analyzed between control and mutant mice.

**Figure 5:**
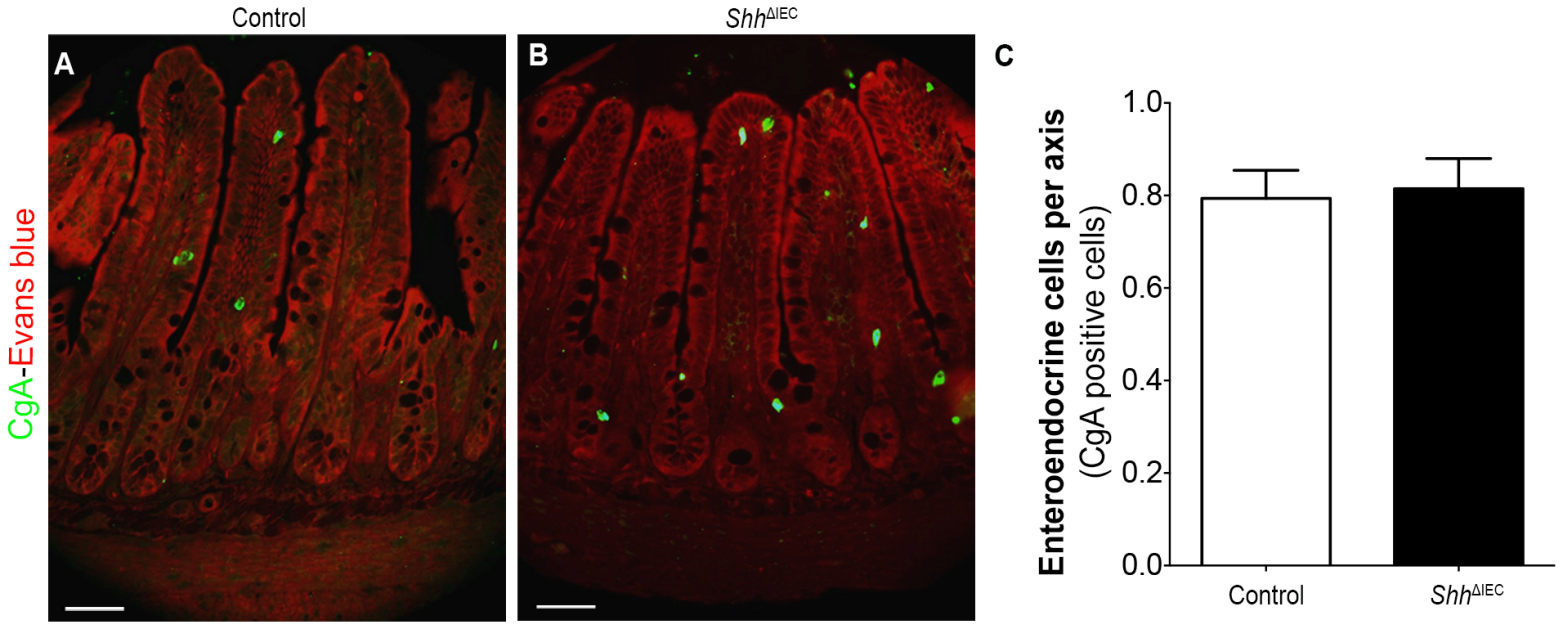
Enteroendocrine cells are not affected by the loss of intestinal epithelial Shh signaling. Chromogranin A was used as a general endocrine cell marker as it labels all subtypes of enteroendocrine cells. No modulation was observed in chromogranin A (CgA)-positive cells (green staining) in the ileal epithelium of control (A) and *Shh* ^ΔIEC^ mice (B). Evans blue (red staining) served as a counterstain. The number of enteroendocrine (CgA)-positive cells was determined from the ileum of control and mutant mice (n = 4) (C). Scale bar: 50 µm. Two-way ANOVA. Error bars represent SEM.

Finally, we characterized if changes in the differentiation and maturation of enteroendocrine cells were observed following the loss of epithelial Shh signaling. Again, no modulation in the number of chromogranin-positive enteroendocrine cells per crypt/villus axis was noted between control and mutant animals (Figures 5A–C).

### Loss of Intestinal Epithelial Shh Signaling Leads to Alterations in the Maturation and Functionality of Intestinal Secretory Paneth and Goblet Cells

**Figure 6:**
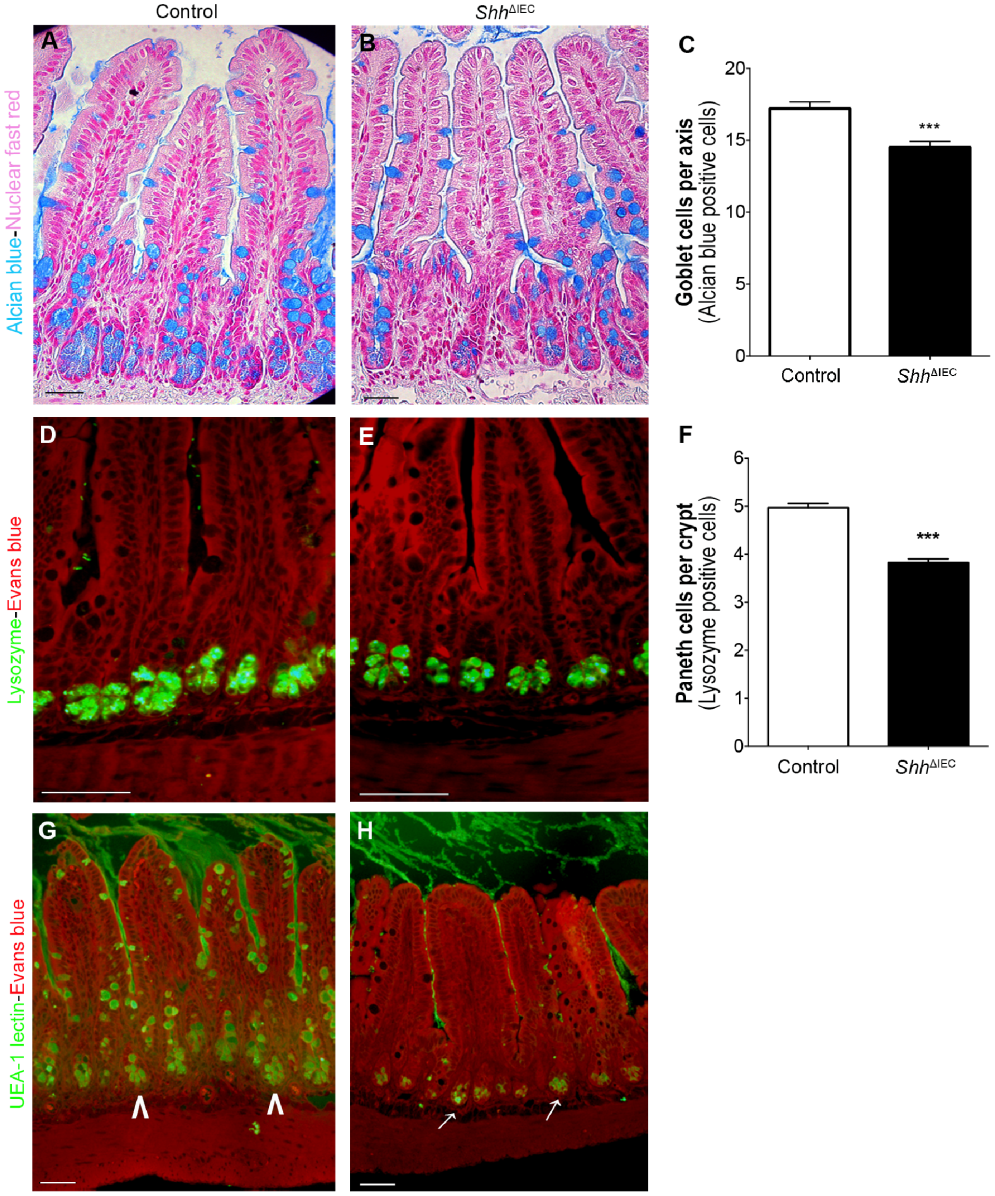
Shh signaling is required for proper production of goblet cell secretory products and Paneth cell maturation. Alcian blue staining was performed to detect mucin (Muc2)-containing goblet cells in control (A) and mutant (B) littermates. Goblet cells were counted from ileum of control and *Shh* ^ΔIEC^ mice (n = 4) with statistical analysis showing a significant 1.2-fold decrease in the number of acidic mucin-positive cells in mutant mice compared to controls (C). Immunostaining with an anti-lysozyme antibody showed a decrease in positively-labeled lysozyme cells (green staining) in mutant mice (E) compared to control littermates (D). Evans blue (red staining) served as a counterstain. Paneth cells were counted from ileum of control and *Shh* ^ΔIEC^ mice (n = 4) and statistical analysis showed a significant 1.3 fold decrease in the number of lysozyme-positive cells in mutant mice compared to controls (F). Fucosylated residues in goblet and Paneth cells were analyzed using Ulex europeus-I agglutinin (UEA-I) lectin staining (G and H). UEA-1 staining exposed an important defect in fucosylation in ileal goblet cells in *Shh* ^ΔIEC^ mice (H) when compared to controls (G), whereas fucosylation was not modulated in Paneth cells of *Shh* ^ΔIEC^ mice (white arrows, H) when compared to controls (white arrowheads, G). Scale bar: 50 µm. Two-way ANOVA ***p<0.001. Error bars represent SEM.

Goblet cells, one of the major secretory cell types of the intestine [14], secrete high-molecular-weight glycoproteins called mucins which represent the main structural components of the mucus layer. MUC2, the major mucin produced by goblet cells [41], was visualized herein with alcian blue staining. A significant 1.2-fold decrease in the number of acidic mucin-positive cells was observed in the ileum of *Shh* ^ΔIEC^ mice when compared to controls (Figures 6A–C).

Another important secretory cell type of the intestine which shares a common precursor with goblet cells are Paneth cells [13], [14]. These cells are known for their important role in stem cell maintenance [42] as well as their antimicrobial role in peptide secretion [14]. Immunostaining against lysozyme showed a significant 1.3-fold decrease in the number of Paneth cells in the ileum of *Shh* ^ΔIEC^ mice when compared to controls (Figures 6D–F).

One important post-translational process that confers the integrity of the mucin polymer is the glycosylation of oligosaccharides [43]. The addition of glycosyl residues to terminal mucin oligosaccharides confers additional resistance to degradation [43], [44]. Ulex europeus-I agglutinin (UEA-I) lectin is used to detect the expression of fucosylated residues on glycoproteins [45]. UEA-1 staining herein exposed an important defect in fucosylation in ileal goblet cells in *Shh* ^ΔIEC^ mice (Figure 6H) when compared to controls (Figure 6G). However, fucosylation was not affected in *Shh* ^ΔIEC^ ileal Paneth cells (Figure 6H) when compared to controls (Figure 6G).

### Loss of Intestinal Epithelial Shh Signaling Leads to a Decrease in IEC Autophagy

**Figure 7:**
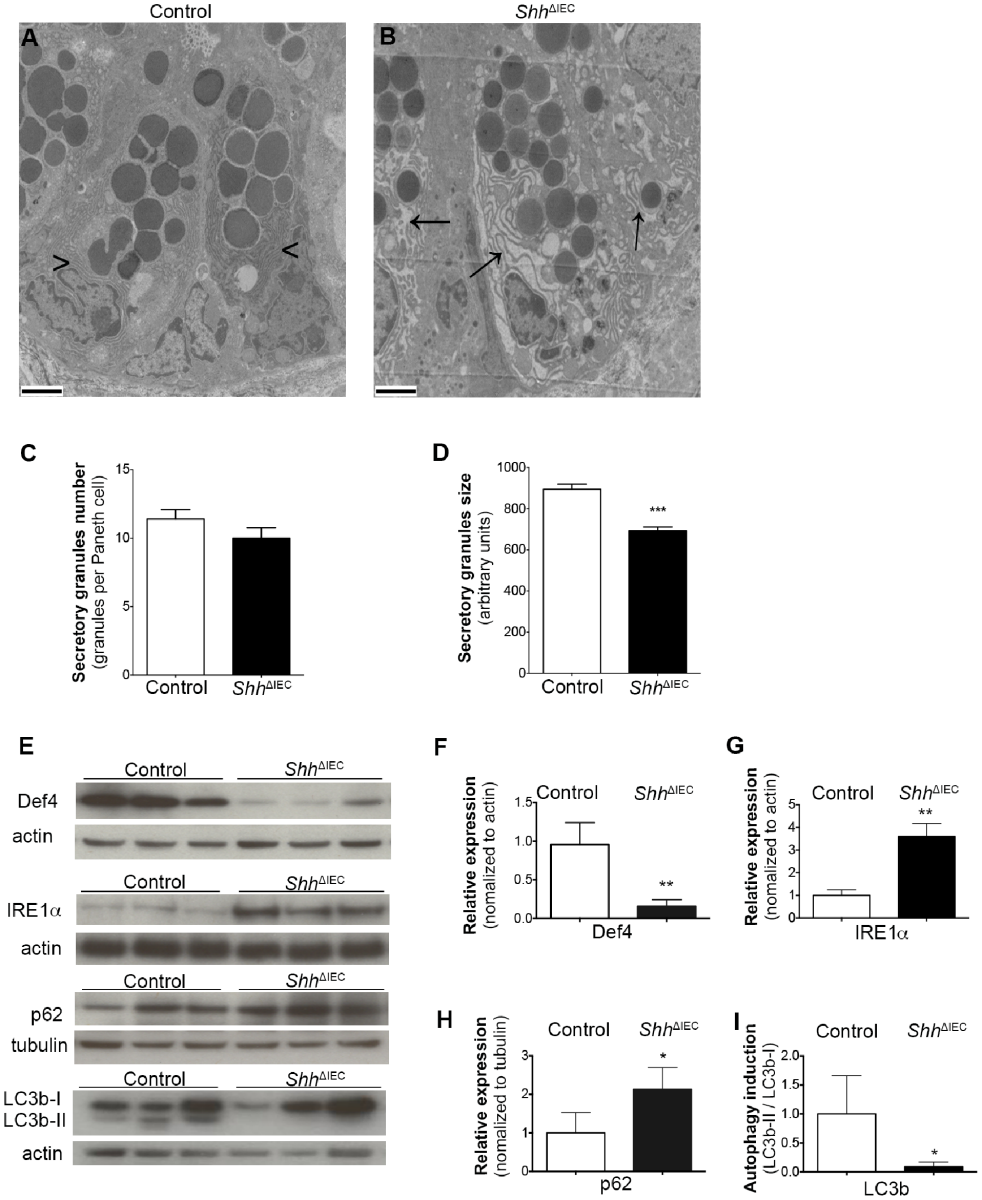
Loss of intestinal epithelial Shh signaling leads to an increase in ER stress and a decrease in autophagy in intestinal epithelial cells (IECs). Transmission electron microscopy revealed a dilated ER lumen in *Shh* ^ΔIEC^ Paneth cells (black arrows, B) when compared to controls (black arrowheads, A). Ultrastructural examination revealed granule abnormalities in mutant mice (B). Paneth granules were counted in both *Shh* ^ΔIEC^ and control mice and no significant modulation was found in the number of granules per Paneth cell between control and mutant mice (C) (n = 4). The mean size of Paneth cell granules was determined using MetaMorph software and statistical analysis revealed that granules were significantly smaller in *Shh* ^ΔIEC^ ileum when compared to controls (D) (n = 4). Western blot analysis against defensin 4, IRE1α, p62, lipidated LC3b-II and non-lipidated LC3b-I proteins were performed on ileal extracts from control and *Shh* ^ΔIEC^ mice (E). Densitometry analysis of exposed films using ImageJ revealed a significant 3.85-fold decrease in defensin 4 expression (F) a significant 3.6-fold increase in IRE1α (G) and a significant 2.13-fold increase in p62 (H) in *Shh* ^ΔIEC^ mice compared to controls (n = 3). An 11-fold decrease in lipidated LC3b-II to non-lipidated LC3b-I ratio in *Shh* ^ΔIEC^ mice can be seen compared to controls (I) (n = 3). Two-way ANOVA ***p<0.001 (C–D). Student t-test *p<0.05, **p<0.01 (F–I). Error bar represents SEM. Scale bar: 2 µm. Def 4, defensin 4.

Highly secretory IEC lineages, namely mucin-secreting goblet cells and antimicrobial peptide-secreting Paneth cells, are vulnerable to ER stress and are dependent on a proper unfolded protein response (UPR) for their homeostasis and function. Sustained ER stress and UPR response may lead to reduced secretory functions or even loss of these functions as a consequence of cellular apoptosis or autophagy induction, in addition to potentially affecting intestinal tissue homeostasis [21], [23], [46]. Since a significant reduction in the number of goblet and Paneth cells was observed in *Shh* ^ΔIEC^ mice without any modulation in apoptosis, we next investigated whether loss of Shh signaling could be linked to ER stress and/or to an autophagy defect. Transmission electron microscopy revealed a dilated ER lumen in *Shh* ^ΔIEC^ Paneth cells (Figure 7B) when compared to controls (Figure 7A). Since the cytoplasm of autophagy-deficient Paneth cells has been reported to contain granule abnormalities [21], [47], we therefore quantified Paneth granules in both *Shh* ^ΔIEC^ and control mice and found no significant modulation of granule number per Paneth cell between control and mutant mice (Figure 7C). However, granules of mutant Paneth cells were significantly smaller when compared to control Paneth cells (Figure 7D). Moreover, these secretory granules store various small molecular-weight antimicrobial proteins, including a variety of α-defensins and lysozyme [48]. Western blot analysis showed a significant 3.85-fold decrease in defensin 4 expression in *Shh* ^ΔIEC^ murine ileal fractions when compared to controls (Figures 7E and F). A dilated ER lumen often suggests an ER stress response [22], [49], [50]. Western blot analysis showed a significant 3.6-fold increased expression of IRE1α, an important marker of ER stress, in *Shh* ^ΔIEC^ ileal epithelium when compared to controls (Figures 7E and G). Interestingly, Western blot analysis also showed a significant 2.13-fold increase in p62 (Figures 7E and H) and an 11-fold decrease in lipidated LC3b-II to non-lipidated LC3b-I ratio in *Shh* ^ΔIEC^ murine ileal fractions when compared to controls (Figures 7E and I). Since accumulation of p62 and the LC3b-II/LC3b-I ratio both represent crucial hallmarks of autophagy [51], our results indicate that intestinal autophagy is impaired following IEC-specific Shh signaling loss.

## Discussion

In recent years, the Hh signaling pathway has been shown to play key roles in gut morphogenesis, cell fate and adult homeostasis [2], [7], [16]–[20]. Despite increasing interest in gut Hh signaling, very little is known regarding the specific roles played by Shh in intestinal epithelial cell function and homeostasis. Herein, we were able to reveal an unsuspected role for Shh signaling in controlling ileal autophagy as well as ileal secretory cell functions, both physiological processes involved in gut homeostasis. In addition to a significant decrease in the number of ileal goblet and Paneth cells upon specific impairment of Shh signaling in the intestinal epithelium, loss of Shh expression led to ER dilation reminiscent of ER stress in ileal secretory cells. Further analysis revealed an increase in ileal p62 and a decrease in LC3b-II, both known hallmarks of autophagy.

Over the past years, studies involving the Hh pathway in the GI tract have either mainly focused on the specific ligand Ihh or have not made any explicit distinction between the various ligands [1], [5], [8], [9], [17], [18], [20], [52]. Part of the reasons for not specifically addressing the Shh ligand were because of the controversy surrounding its expression in the intestine and, as a result, Ihh was ultimately considered to represent the main Hedgehog ligand expressed in the intestine [1], [5], [8], [17], [18]. Our data confirmed that Ihh was expressed along the entire intestinal tract of adult mice. However, we also confirmed that Shh was significantly expressed by the crypt epithelial cells in both the ileum and proximal colon comparatively to the most proximal sections of the small intestine. Coincidently, conditional deletion of *Shh* in the intestinal epithelium was found to only significantly affect the ileum. The fact that phenotypic changes were not observed in the proximal colon of *Shh* ^ΔIEC^ mice, despite detection of significant endogenous Shh expression, could be explained by possible partial Shh deletion in our experimental conditions. However, our data strongly support that Shh may play a distinctive role from Ihh in the distal section of the small intestine.

Ubiquitous inactivation of the Shh ligand has been shown to result in specific neonatal gut phenotypes in mice [4]. Indeed, *Shh* mutants exhibit a hyperplastic stomach, duodenal obstruction, increased epithelial proliferation and abnormal innervation of the gut [1], [3], [4]. Surprisingly, we observed an opposite phenotype in the adult with a decrease in ileal epithelial proliferation. However, opposite phenotypes between embryonic and adult stages have previously been observed with regard to morphogens including Hh [9], [18]. Indeed, whereas in the developing gut, Ramaloh-Santos et al. reported that *Ihh* mutants displayed reduced epithelial stem cell proliferation and differentiation [4], conditional gut deletion of *Ihh* revealed an opposite phenotype in the adult gut [18]. Two major transitions occur from the time of endoderm delineation at gastrulation until establishment of adult gastro-intestinal morphology and function [15]. Beginning at embryonic day 14.5 in the mouse, there is a morphological transition from a stratified epithelium to a simple columnar polarized epithelium. At this stage, the underlying mesenchyme invaginates into the epithelium inducing the formation of epithelial ridges, which ultimately give rise to the villi of the mature intestine. The second transition occurs at weaning, at about postnatal day 18–22. At this time, there is a profound change in the pattern of gene expression that leads to the adult phenotype and functionality of gut organs [36]. In this study, we used the non-inducible transgenic *Villin*Cre line that drives Cre expression in the intestinal and colonic epithelium starting at E15.5 [24], [53]. Thus, deletion of *Shh* in the current mouse model encompassed only the late embryonic stage and adult life of the animal unlike the ubiquitous inactivation of the Shh ligand that cover all stages. Although we cannot exclude a role for a late embryonic window in some of the phenotypes observed in the *Shh* ^ΔIEC^ mice, we believe our observations in the adult mouse ileum could suggest a similar opposite paradigm for Shh between fetal and adult gut tissues.

Recent studies on the impact of Ihh on adult gut tissue have proposed this specific ligand to be detrimental for mature absorptive cell maturation and function, thereby benefiting the secretory cell lineage [9], [18]. Our current study revealed that deletion of Shh had no impact on absorptive cell specification, maturation and function. A similar finding was observed with enteroendocrine cells from the secretory lineage. The only epithelial cells affected by the loss of Shh signaling were Paneth and goblet cells in the ileum, where a significant reduction in their respective cell numbers was observed. Ultrastructural microscopic analysis also showed a dilated ER lumen in *Shh* ^ΔIEC^ Paneth secretory cells. Dilated ER lumen often suggests an ER stress response resulting in the accumulation of unfolded proteins in the ER lumen and the induction of the unfolded protein response (UPR) [22], [49], [50]. The increased expression of IRE1α in *Shh* ^ΔIEC^ ileal epithelium observed herein confirmed the induction of ER stress following loss of the Shh ligand. This is in keeping with the fact that highly secretory IEC lineages, such as mucin-secreting goblet cells and antimicrobial peptide-secreting Paneth cells, are vulnerable to ER stress and are dependent on a proper UPR for their homeostasis and function [21], [54]. Sustained ER stress and UPR response may lead to reduced secretory functions or even loss of these functions as a consequence of a reduction in cellular autophagy. Indeed, autophagy is a cellular process by which different cytoplasmic components including organelles are targeted for degradation to the autophagosome. Autophagy can be a protective mechanism and could counterbalance the expansion of ER following disruption of UPR [50], [55]. Down-regulation of autophagy has furthermore been shown to negatively influence intestinal epithelial cell secretory functions [21] and may affect intestinal tissue homeostasis [21]–[23]. Interestingly, recent evidences showed that Shh signaling was able to activate autophagy in various cellular origins including hippocampal neurons, hepatic cancer cells and vascular smooth muscle cells [56]–[59]. Given the fact that several alterations observed herein in *Shh* ^ΔIEC^ mouse ileum share many similarities with recent reports on intestinal secretory cells defective for autophagy, together with the observed important reduction in LC3b-II and an increase in p62 in this context, we propose that Shh signaling could be similarly important in supporting IEC autophagy in the ileum.

In summary, the present data are the first to demonstrate that epithelial Shh, as an Hh pathway ligand, plays a specific role in the intestinal ileum. Most importantly, we demonstrate that the loss of Shh in the intestinal epithelium limits ileal secretory cell functions and epithelial autophagy. Autophagy proteins are required for secretion and subsequent loss of these proteins results in abnormal secretory functions. Given that delivery of granule content to epithelial surfaces by secretory cells is a critical physiological process, addressing the specific role of Shh signaling in the context of ileal physiopathology should be of key interest in future studies.
